# Equalisation of Children‘s Various Levels of Physical Activity Using Increased Physical Activity at School Among Ninth Graders

**DOI:** 10.3389/fpubh.2022.856794

**Published:** 2022-04-27

**Authors:** Hege Hov Lomsdal, Sondre Arntzen Arntzen Lomsdal, Pål Lagestad

**Affiliations:** Department of Teacher Education and Art, Nord University, Levanger, Norway

**Keywords:** physical activity, school, children, accelerometer, intervention

## Abstract

The purpose of this study was to examine whether 60 min of physical activity implemented during school hours, would have an impact on 15-year-olds' MVPA (moderate to vigorous physical activity) throughout the school day, and what effect it would have on low-, average-, and high- physically-active students. The intervention study included a sample of every 93 students in the ninth grade from a school in Trøndelag, Norway. Data on the students' physical activity levels during schooltime were measured using accelerometers and analyzed using the Actilife program. A significant higher change in MVPA was found in the intervention group compared to the control group, with an average increase of 25 min in MVPA in the pre-test to 42 min in post-test. Further analyses showed that the that both the low-active and the high-active had a significant increase in MVPA, taking the results of the control group into consideration. However, the low-active participants had the largest increase, with a 123% increase in MVPA during schooltime. The implication of the study is that PA interventions in school have the largest percentage effect among the low-active students in the study, which indicates that school-based interventions can be important in bridging social differences in MVPA among adolescents.

## Introduction

Today's society is characterized by inactivity and sitting still more than before, and physical inactivity has, in a long-term perspective, a negative effect on children and young people, bringing disadvantages from both a public health and a socio-economic perspective (1, 2). The positive effect of physical activity (PA) for children and young people has led, in recent years, to many interventions focusing on raising young people's activity level, and school has been an important arena for such interventions and measures (3).

International (and Norwegian) health recommendations say that children and young people should engage in PA for a minimum of 60 min moderate to hard intensity (MVPA) each day (2, 3). The results from recorded measurement of the PA of Norwegian 6-, 9- and 15-year-olds show clear signs that the level of activity is too low in relation to the recommendation (4–6). Among 15-year-olds, only 40% of the girls and 51% of the boys met the recommended level (4). Studies indicate that the level of adolescents' PA has been relatively stable in recent years, but with a small downturn between 2005 and 2018 (4–6). Longitudinal studies have pointed to a decrease in children's and young people's activity during their adolescent years (4, 7, 8). On a world basis, it is estimated that more than 80% of young people get insufficient moderate to hard PA (9).

The falling level of PA has, in light of the consequences it brings, led to an increased focus on PA and health promotion work in the western world, with school being seen as a particularly suitable arena for interventions to increase children and young people's activity level (10–12). School's infrastructure is very well suited to trying out such interventions (1, 11, 13), and given that school (as opposed to sport) is a place where all children can be reached, it is particularly well-suited, in terms of adolescents' level of PA, to leveling out social disparities. A central and underlying aim of Norwegian education policy is that school should have a leveling effect on society: it is about reducing the differences and inequalities between the various classes in society (14). Social leveling means that the possibility of succeeding will be the same irrespective of one's family background. In this way, school will not necessarily create equal students, but rather give all students an equal opportunity for both learning and development. Several studies have seen a positive correlation between socio-economic status, and children and young people's level of activity (15–17). By using school as an arena for intervention to raise the level of PA among children and young people, all children are reached, and their socio-economic status does not need to be a crucial factor (11). Another important, positive factor of school-based interventions is that the total time spent at school is continuous over a long period, which is relevant and importance when an intervention takes place (18). Trudeau and Shepars (19) see school as an important arena for achieving greater PA in line with the recommendations and argue that incorporating PA into the theoretical subjects does not adversely affect academic performance.

The USA is one of the countries with a clear recommendation in relation to school's accountability in relation to children's total PA. The recommendation is that school take responsibility for activating the children for 30 of the recommended minutes of activity (20). In Norway, on the other hand, there is, for the moment, no comparable recommendation regarding school's responsibility, but in 2017 a proposal, which received majority assent, was put before parliament in which the proposers referred to a minimum of 1 h PA each day for everyone in grades 1–10. The proposal read as follows: “Parliament begs the government to introduce a measure guaranteeing pupils in grades 1–10 at least an hour's PA each day and within the school timetable, and that this be funded as a public health measure.” (21). The proposers used as a basis the well-documented connection between health and PA, with the activity level in Norway being considered to be low, as with other Western countries. The call was that the hour be planned, adapted and led by a competent teacher (21).

Many intervention studies have been conducted with the intention of increasing knowledge about PA. Most have been directed toward adults and children, while studies aimed at adolescents are largely absent (1). This is paradoxical when one considers that it is precisely in adolescence that PA falls away (often related to “drop-out” from organized sport), and that it is during this period that most people form habits that they often maintain throughout the rest of their life. Kristiansen et al. (22) found that schooltime for 12–13-year-olds accounted for 31 and 26% respectively of boys' and girls' total weekly MVPA, with a mean of 13 minutes MVPA. Andersen (23) found a mean MVPA of 19 min during schooltime. It is very important that the least active increase their activity.

A number of intervention studies into increased PA in school have been conducted. In Active Smarter Kids, 60 min a day of teacher-led PA was implemented. The effect, which was measured as aerobic endurance, proved to be considerable, leading to the school management continuing to use the model (24). The effect of such interventions has, however, shown itself to be variable, which may be a result of methodology and strategies during the period of intervention (24–26). Resaland et al. (24) have, in addition to sound planning and organization, pointed to communication, supervision and teacher training as important factors in the eventual success of an intervention. Tillaar et al. (26) mention lack of significant follow-up, procedures and organization as being possible reasons for lack of effect. Inchley et al. (27) argue that the implementation of PA in schools should receive greater recognition to achieve the desired changes. School management, parents and teachers should be both motivated and engaged in implementing, preferably over a longer time, the measure designed to raise the pupils' level of activity (28, 29).

On the basis of the above discussion, this study will look closely at the effect of an intervention intended to implement 60 min of PA every day during schooltime. The research question is as follows: What effect will the implementation of 60 min PA, as a part of school's academic curriculum, have on 15-year-olds' MVPA in schooltime, and to what extent will such an intervention even out differences among students with different activity levels?

## Methods

The study uses data from a larger study concerning the introduction of daily PA in middle school, and where, also, pupils' activity levels were measured by accelerometer. These measurements took the form of a pre-test and a re-test. In advance of the project, approval was granted by the Norwegian Centre for Research Data for data collection to be carried out. Both pupils and teachers gave their assent to participation in the study, and the pupils and their parents gave written consent.

### Participants

The participants in the study came from a middle school, chosen by means of a stratified selection, in Trøndelag county. The school had four 9th grade classes, consisting of 93 pupils (14 years of age) and, in all, 12 teachers. 91 pupils had valid accelerometer data. One of the four classes was randomly selected as a control group (*N* = 21, 12 girls and 9 boys). This class was used as a control group throughout the entire project. The other three classes carried out the intervention and became an intervention group (*N* = 70, 38 girls and 32 boys).

### Procedures

Ahead of pre-testing, all the pupils received instruction from the project leader (one of the authors) in the use of the activity meter. The training included guidance on where it should be placed, when it should be on, and they were able to try out for themselves how to attach and place it. This was done to ensure correct use of the equipment. Pre-testing was carried out during a 2-week period (10 teaching days), during which both the control group and the intervention group received their normal teaching with their 12 teachers. Re-testing was carried out during a 2-week intervention period (10 teaching days), during which the intervention group was offered a teaching programme aimed at 60 min of PA each day, while the control group had their normal teaching. The measures that were implemented in everyday school life were PA used as a teaching method within traditional academic subjects, such as languages and mathematics. During the intervention period, the 12 teachers were to implement PA as part of the academic content of their teaching using a strategy called physical active learning (PAL), which falls under what is known as “movement integration” (30), implying that PA is implemented in regular teaching hours, in the classroom or elsewhere (31). Research related to “movement integration” has led to positive results related to increasing children's MVPA at school (12, 32, 33). During this intervention period, more time was given to PA by reallocating time within other subjects. Sixty minutes of PA were carried out each day, the sessions being at class level, and led by the same 12 teachers as in the control period. The strategy involves PA being incorporated into the normal lessons, in the classroom or elsewhere (31). The subjects involved was Norwegian, Mathematics, Science, Social Science, Arts, English and Christianity. The sessions were at class level, and teacher led. The teaching plan was put together and discussed jointly, with guidance from one of the authors. The emphasis during this period was active learning, with the teacher using PA as a way of communicating the material in the theory lesson.

### Data Collection

Level of PA was measured using Actigraph GT1M (ActiGraph LLC, Pensacola, FL, USA) accelerometers. ActiGraph is known to be highly effective, and is widely recognized in studies related to PA (34, 35). Migueles et al. (36) show that more than 50% of all published articles of PA have used Actigraph in their studies to acquire objective measures of PA.

On initialization, an EPOCH-length of 60 s was chosen. This little meter records the student's PA (13). The same type of accelerometer, as well as procedures used in this study, were deployed in line with procedures from the largest population studies done on measurements of children and adolescents' degree of activity (4, 6). All movement to which it is exposed is registered, and all activity outside normal human movement filtered out (6). The activity monitor registers acceleration, which is converted into digital signals, known as “counts”. These counts describe the level of acceleration that the accelerometer itself was subject to. A low level of counts per minute indicates a low average level of activity, while a large number of counts per minute indicates a high average level of activity. These counts are recorded continuously and saved at a pre-set time interval (epoch). This time interval is usually between 5 and 60 s (37). The limit value used in the analysis of moderate intensity was set, in this study, at 2000 counts, in line with other, large Norwegian surveys (6). In this way, activity during schooltime can be isolated, so that the actual activity level of the participants at different points in the school day was measured. This can contribute to a clearer picture of children and adolescent's level of activity in schooltime, break periods and physical education (38).

According to the protocol, each individual pupil should have had the accelerometer on them for two consecutive weeks (10 school days), in two periods. In line with the procedure (6), the pupils placed their accelerometer on their right hip each morning as school started and took it off at the end of the school day. School activity was isolated, so that the actual activity level of the students was measured. The teachers, throughout the project, were responsible for handing out and collecting in the accelerometers. The teachers had their own suitcases, which were marked each with their own number for each student. In this way, errors were avoided, in which, for example, the wrong tape was delivered to the wrong person. Training in the use of the accelerometers was introduced in advance of the intervention period, to ensure that the equipment was used correctly. The project leader was present during this period and during the first 2 days of the control period. This approach increases reliability. After the 14 days (10 school days), the accelerometers were collected in, and the data downloaded in the program Actilife, and analyzed. The same procedure was repeated after the 2 weeks of the intervention period.

### Statistical Analyses

On the basis of the pre-test measurements from the control period, the pupils were categorized into three groups based on the MVPA values; low-active: 0–20 min, medium-active: 20.01–30 min and high-active: 30.01–50 min. The statistical analyses performed were made using the statistical program SPSS version IBM SPSS 27. The descriptive statistics used in this study are presented as average and standard deviance (SD). To evaluate the difference in size of the changes between the control and intervention groups (post-test minus pre-test), independent *t*-tests were used, and to explore the changes between pre- and post-tests, paired sample t-tests were used. The statistical significance was set at < 0.05.

## Results

### Effect on MVPA of the Introduction of 60 min PA

**Figure 1:**
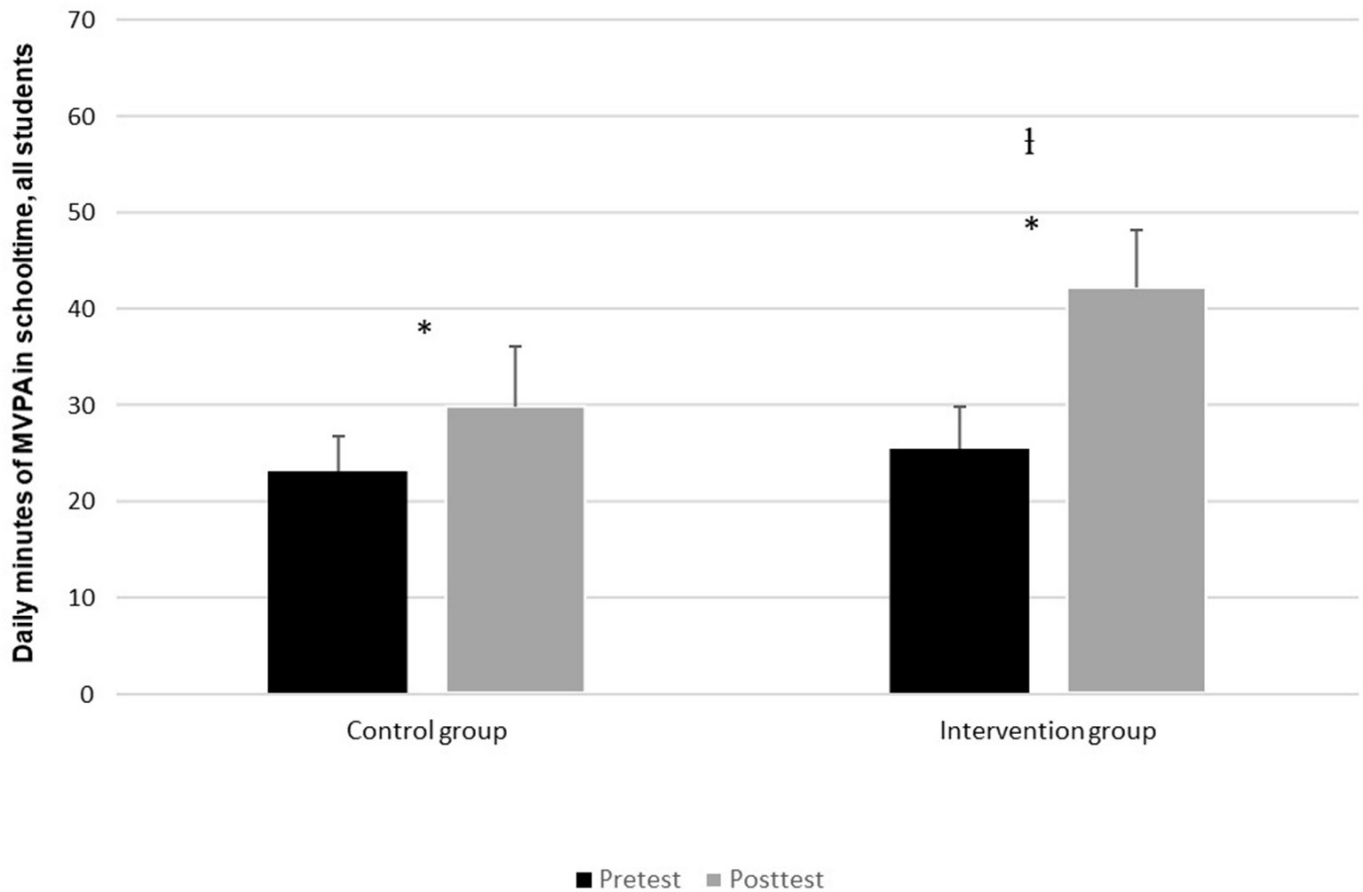
Average daily contribution of MVPA (min) in school time at pre-test and post-test. *Indicates a significant difference (*p* < 0.05) between pre-test and post-test in daily MVPA in school time. ^I^Indicates a significant difference (*p* < 0.05) in MVPA development between the control group and the intervention group.

In Figure 1, the daily contribution of MVPA during school time (minutes) is presented for the control group and the intervention group, tested by both pre-test and post-test of the pupils. The results show that the control group reached 23 min MVPA during the school day at pre-test, and 30 min at post-test. This is an increase of 30%. In the intervention group's case, they reached an average of 25 min at pre-test, but post-test showed an average of 42 min, a 66% increase. The Figure shows that the increase from pre-test to post-test was significant for both the control group (*t* = −4.2, *p* < 0.05) and the intervention group (*t* 0–16.8, *p* < 0.05), but the intervention group had a significantly higher increase than the control group in this period (*t* = −4.2, <0.05).

### Changes in MVPA for the Low-, Middle and High-Active

**Figure 2:**
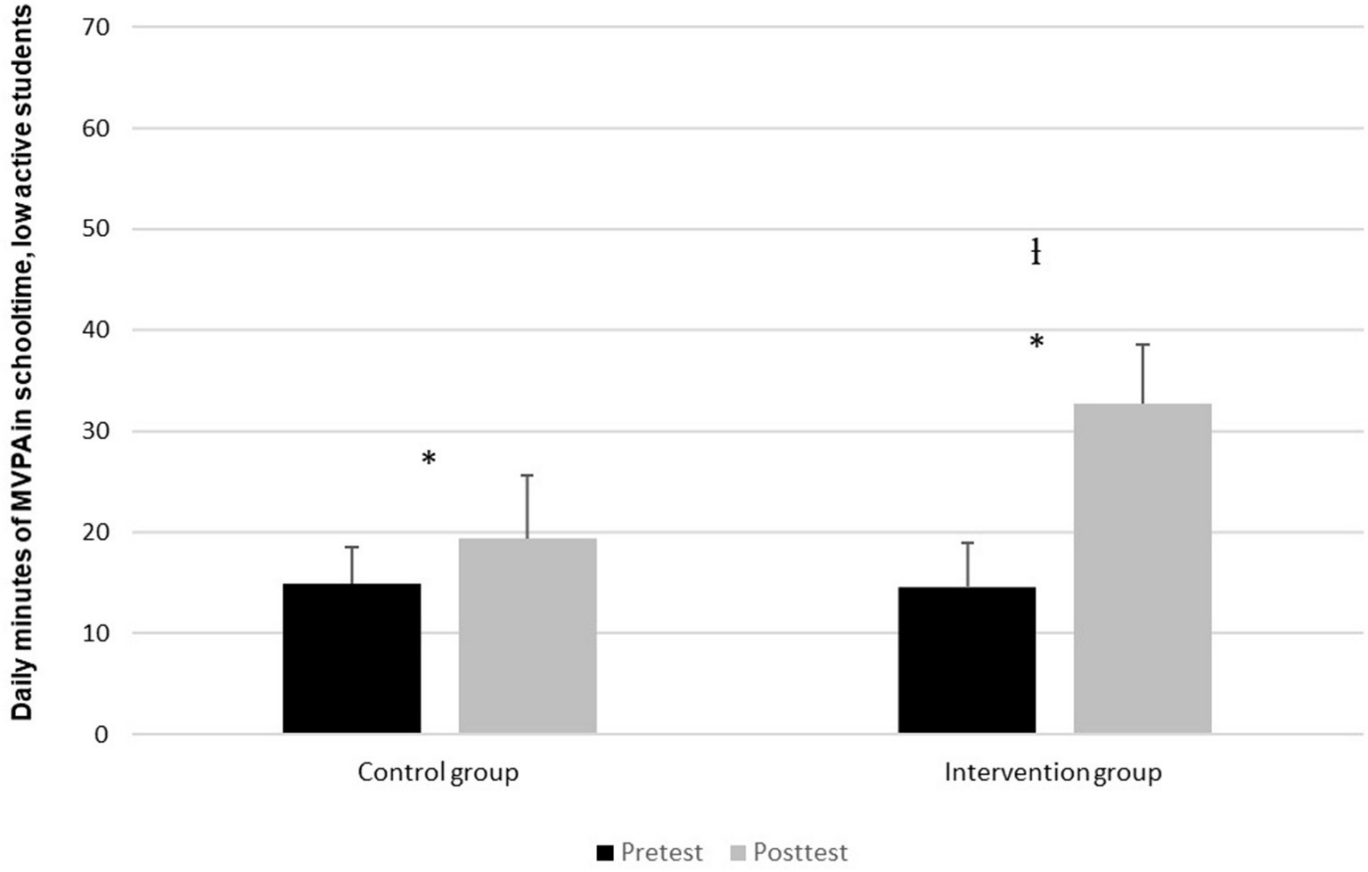
Daily contribution of MVPA in schooltime for pupils in the low-activity group, divided between pre-test and post-test. *Indicates a significant difference (*p* < 0.05) between pre-test and post-test in the daily MVPA during schooltime. ^l^Indicates a significant difference (*p* < 0.05) in MVPA development between the control group and the intervention group.

Figure 2 shows that the average MVPA per day in schooltime among the low-active in the intervention group is significantly higher in the post-test than in the pre-test (*t* = −15.9, *p* = <0.05). The percentage change here is 123% based on the results from the pre-test which showed an average of 14 min, and a post-test average of 32 min. In the control group there is also significant change from pre-test to post-test (*t* = −4.6, <0.05). The pre-test showed an average of 15 min, giving a percentage-wise change of 31%. The change in the intervention group is, however, significantly greater than the control group (t = −7.9, *p* <0.05).

**Figure 3:**
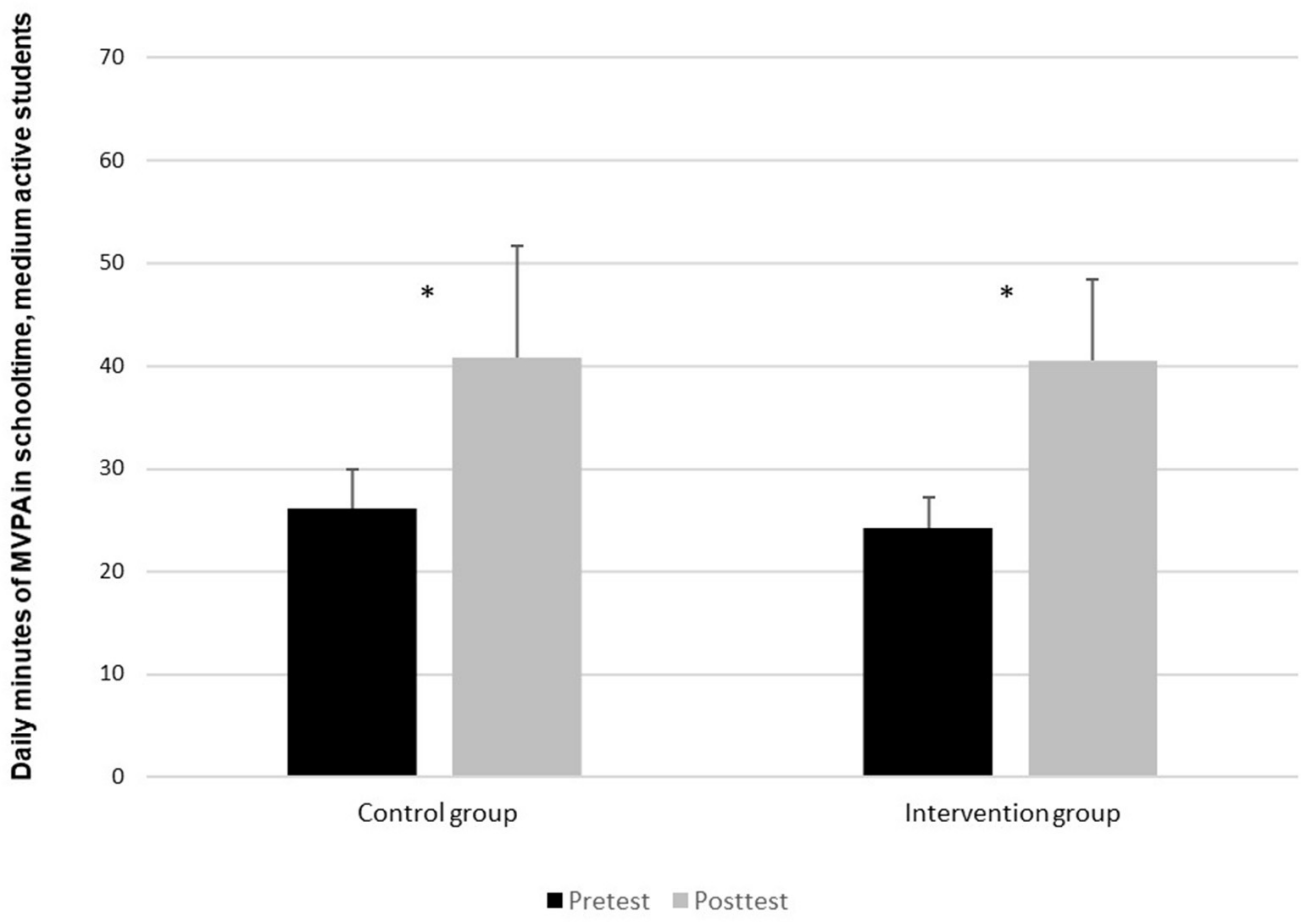
Daily contribution of MVPA in schooltime for pupils in the medium-active group, divided between pre-test and post-test. *Indicates a significant difference (*p* < 0.05) between pre-test and post-test in daily MVPA in schooltime.

In Figure 3, one also finds a significant increase among pupils in the intervention group categorized as medium-active (*t* = −10.3, *p* < 0.05), with a 67% increase from the pre-test average measurement of 24 min and the post-test measure of 40 min. There was also a significant difference in the control group (*t* = −4.2, p < 0.05). The pre-test in this group showed an average of 26 min, while the post-test showed 40 min, amounting to a difference of 56%. There was however no significant difference between the change in the control group and the intervention group (*t* = −0.4, *p* = 0.07).

**Figure 4:**
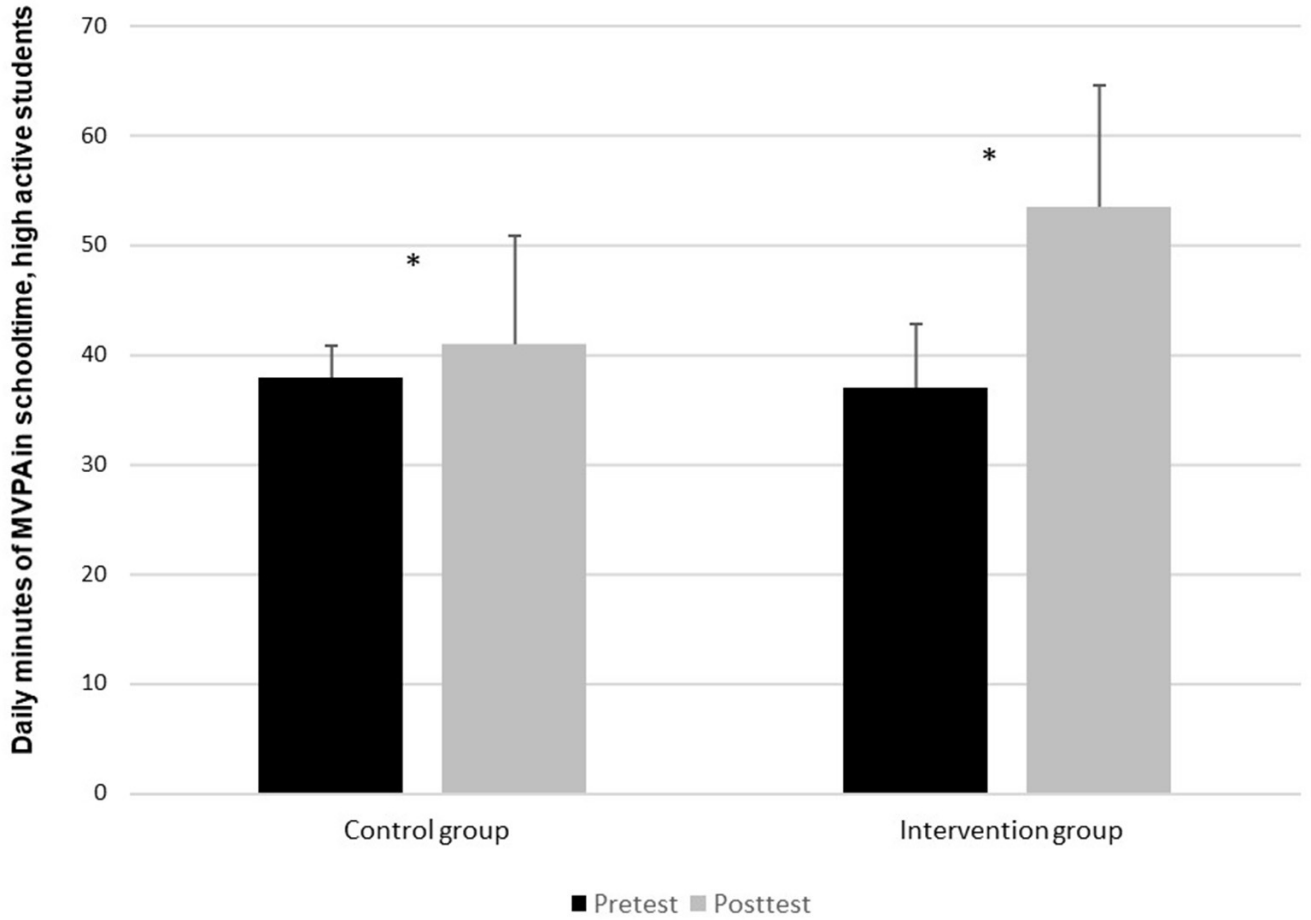
Daily contribution of MVPA in schooltime for pupils in the high-active group, pre-test and post-test.*Indicates a significant difference (*p* < 0.05) between pre-test and post-test in the daily MVPA in schooltime of the intervention group.

Figure 4, like the other figures, shows a significant increase in the intervention group from pre-test to post-test (*t* = −7, *p* = < 0.05), with a percentage increase of 44% from pre-test to post-test. In the control group, however, there was no significant difference between pre-test and post-test (*t* = −1, *p* = 0.391).

## Discussion

### Effect on MVPA of Implementation of 60 min PA in School

The results have shown that 2 weeks with daily implementation of 60 min PA in different academic subjects led to a significant increase in the pupils' PA, where the intervention group had a significantly higher change in PA compared to the control group. The total percentage increase was 66% from pre-test to post-test in total MVPA for the intervention group, compared to the control group where the increase pre-test to post-test was 30%. As with Larsen et al. (39), the intervention appears to have been successful in our study. Larsen et al. emphasize that the pupils in their study had an extremely positive experience from the combination of academic studies and PA, finding this a highly motivating and stimulating combination. Our findings support earlier studies which have shown school to be an arena well-suited to testing different implementations (10–12, 20).

At the time of writing, it is 5 years since a majority in Parliament supported a proposal that all children have the opportunity to have 1 h PA at school (21), but the measure has still not come into effect on account of, among other matters, a lack of research, together with questions concerning its cost. This research, in common with that done earlier into children and young people's PA in school (24) is indicates strongly that the initiative has a positive effect on pupils. Research has shown that PA among children and young people affects their social life and represents an important prerequisite for them being able to master both school and life in general (40). Against this background, there is reason to think of the time spent at school as being especially important for children and adolescents as it gives them the possibility of learning good lifelong habits regarding PA, which, in that way, represents a good long-term investment. And, when it comes to finance, there is also good reason to believe that inactivity will carry a greater cost in the long run. The positive results from this study also reinforce the previously mentioned research indicating that implementation of PA in schools ought to be given greater recognition in respect of achieving the desired change (27). An implementation making use of school as an implementation arena can offer pupils a safe environment and mean that all pupils, regardless of differences in motivation for PA, can readily take part (13).

The findings of this study show from the pre-test an average of 25 min of MVPA achieved in school time across all the pupils participating in the study (control group and intervention group). This is somewhat more than in an earlier study of Norwegian 15-year-olds (23), which found an average of 19 min MVPA in schooltime. Kristiansen (22) found an average of 14 min MVPA in schooltime among 12–13-year-olds. These studies included relatively many students from quite many schools. It can appear, therefore, that the school chosen in our study, is a school with relatively physically active pupils.

Against the background of a large percentage of children and adolescents in Norway not fulfilling the recommended healthy level of 60 min of PA per day, the findings in this project can give a clear indication that raising the level of activity at school will also raise children's MVPA level, which is highly desirable from a health perspective. This in the hope of satisfying the health recommendations to a greater extent than previous research has shown young people to be doing at present (4–6). If the recommended American level of 30 min MVPA during schooltime (41), was linked to the results following the intervention in our study, all the pupils would, on average, have attained this level. The results indicate, nonetheless, that despite such an implementation, there will still be some pupils who will fall short of the recommended level of PA. Our findings suggest that school isn't able to assume full responsibility for their pupils' MVPA, and that other things such as sport, leisure activities and, not least, parents and carers, are important in respect of contributing to young people's PA.

### Equalization of Difference in Activity Level Through the Implementation of 60 min PA

The results show the clear positive effect of the intervention, particularly among low-active pupils. The results showed that the low-active in the intervention group had an increase in minutes of MVPA of 123% between pre-test and post-test. The medium-active members of the intervention group had an increase in minutes of MVPA of 67%, while the high-active members of the intervention group had an increase in minutes of MVPA of 44%. There were also significant changes for the low-active and medium-active in the control group. The changes were, however, significantly greater in the intervention group than in the control group. Among the high-active, there was no significant change in the control group, only in the intervention group. From these findings, one can claim that both the low-active and the high-active profit from the intervention, but that the intervention has the greatest effect among the low-active pupils. There are therefore grounds to say that such strategies have a positive effect in equalizing differences in activity level among the pupils. This is beneficial given that research has shown that children from families with low socio-economic status have a lower level of activity (17, 42), and pointed to school as a suitable arena for equalizing differences in PA (5).

Our results have shown that there are big differences among pupils in activity level in schooltime, but that school can succeed in evening these out. By using school as an intervention arena, one is able to reach all the children, irrespective of social inequalities, and contribute, thereby, to reducing these inequalities (43, 44). Using school as an arena for PA interventions ensures that all children have the possibility of PA to a much greater extent than do similar measures involving, for example, sport. Our findings appear to show school to be a highly suitable arena for leveling out differences in MVPA among the pupils. This is in agreement with earlier research (45, 46). These findings suggest there may, therefore, also be grounds for seeing school as a particularly suitable arena for equalizing social inequality in health. Increased PA in schooltime will also, according to Bastian et al. (47), affect the activity level of pupils in their leisure time, which is, again, an important factor in leveling out social inequalities in health in the longer term. This is because school-based interventions, through facilitation and structural measures, also had a positive effect on the activity level of children and young people in leisure time.

Looking at it critically, one can argue that even though the increases in MVPA are large with such an intervention—especially among students in the low-activity group, one is still not reaching the target set by the health recommendation of 60 min daily MVPA (4). At least not if one looks only at the figures for schooltime. For the high-active, we find an average of 54 min MVPA, which should be seen and interpreted as almost achieving the goal set by the health recommendations, The pupils categorized as medium-active achieved an average of 41 min MVPA, while the low-active came out with an average of 33 min MVPA. Previous research has shown that those students who are not so physically active during school time have problems in “catching up” with those who are sufficiently active in their free time, and, in this way, it becomes difficult both to establish good activity habits, and to provide good coping experiences for these children (48).

### Strengths and Weakness of the Study

A very high participant adherence of 98% and 91 students who used the accelerometer for 20 schooldays, can be thought of as a strength. The use of a control group in the intervention as was done here, represents a strength in comparison to studies not using one (13, 49).

An epoch length of 60 s was chosen at the initialization of this study. There is room for discussion as to whether this is a strength or a weakness, but on the basis of previous research it is difficult to either disprove or confirm a “correct” formula. It can be seen as beneficial to have a storage interval of 10 s for children and adolescents due to a sporadic activity level (6, 50), while several studies of adults have used an epoch length of 60 s (51, 52). A Norwegian study of nursery children nonetheless used an epoch length of 60 s (53). There is a considerable difference in activity-rhythm between 6-year-old children and 15-year-olds who have a much less sporadic rhythm, closer to that of adults. On that basis, the choice of an epoch length of 60 s can still very likely be appropriate.

The study also has certain weaknesses. It would have been advantageous to have had more schools in the study, to have had an even larger sample that would have given greater reliability to the study (54). Random sampling would have been more representative, making it easier to generalize the results. Another potential weakness of the method used in this study can be the accelerometers and their limited ability to not register non-ambulatory activities such as climbing, cycling and strength-training (55–59). This may lead to certain activities being overlooked as they are not recorded accurately (37, 56, 58). Also, a further weakness of the accelerometers is that they cannot be in contact with water, leading to swimming not being registered. In the case of this study, neither swimming, nor cycling or strength-training were a part of schooltime during this study. Finally, the inequality of the groups and the small control group is a weakness of the study.

## Conclusion

The results of this study show that the implementation of 60 min PA in schooltime, raises the pupils' MVPA by 68%. This gives a clear indication that school is both an effective and important arena for interventions aimed at increasing children and adolescent's PA. Further, this study finds that implementation of 60 min of PA in schooltime has a positive effect in equalizing differences in activity level among children during schooltime. In the study, we found that it is low-active children who gain the most, relatively, from such an intervention. For this reason, we argue that school is not only an important societal mandate when it comes to equalizing differences in activity in school, but also seems to be a good arena for leveling out the large differences in PA level among children and young people. This is because school, unlike sport, reaches all children, and, not least, that PA interventions like this are found to equalize differences in adolescents' PA levels. Further research should include a larger number of young people, using randomized selection supplemented by qualitative interviews and questionnaires gathering in the pupils' and teachers' reflections concerning PS interventions in school.

## Data Availability Statement

The raw data supporting the conclusions of this article will be made available by the authors, without undue reservation.

## Ethics Statement

The studies involving human participants were reviewed and approved by the Norwegian Centre for Research Data. Written informed consent to participate in this study was provided by the participants' legal guardian/next of kin.

## Author Contributions

HH has written most of the article, especially the introduction, discussion, and conclusion. SL has participated in the design and data collection and has also participated in some of the writing. PL has participated in the design, data collection, analyses of the data, writing the results, has also participated in some of the writing of the introduction, discussion, and conclusion. All authors contributed to the article and approved the submitted version.

## Conflict of Interest

The authors declare that the research was conducted in the absence of any commercial or financial relationships that could be construed as a potential conflict of interest.

## Publisher's Note

All claims expressed in this article are solely those of the authors and do not necessarily represent those of their affiliated organizations, or those of the publisher, the editors and the reviewers. Any product that may be evaluated in this article, or claim that may be made by its manufacturer, is not guaranteed or endorsed by the publisher.
